# Universal screening for postpartum depression? Inequalities and barriers among immigrant mothers

**DOI:** 10.1093/eurpub/ckac129.455

**Published:** 2022-10-25

**Authors:** M Marti Castaner, C Hvidtfeldt, JM Møller Olsen, M Nørredam, S Fredsted Villadsen

**Affiliations:** 1 Health Services Section, University of Copenhagen, København, Denmark; 2 Research Unit, Rockwool Foundation, København, Denmark; 3 Social Medicine, University of Copenhagen, København, Denmark

## Abstract

**Background:**

Postpartum depression (PPD) is a serious public health concern affecting 12% of women globally. Early detection is necessary to provide timely support. In Scandinavian countries, universal screening is recommended. However, migrant women, who are at increased risk of PPD, seem to be screened less often. Still, there is limited knowledge about 1) how acculturation relates to screening, and 2) how healthcare providers navigate PDD screening with migrant women.

**Aims and methods:**

We used a mixed-methods approach to explore whether and why inequalities in PPD screening using the Edinburgh postpartum depression scale (EPDS) may exist in the context of universal PPD screening within the free home-visiting program in Denmark. Data from 77,694 infants and their mothers participating in the Danish home-visiting program (2015-2018) was used to examine the prevalence of participation in PPD screening and its association with migrant status and acculturation factors. We interviewed 16 health visitors to examine qualitatively challenges and strategies used when assessing PPD among immigrant mothers.

**Results:**

Immigrant women were 80% more likely to lack screening (adj. RR 1.81-1.90). All factors indicating lower acculturation, such as shorter length of residence, older age at migration, and having studied abroad were independently associated with increased risk of lack of screening. Thematic analysis of qualitative data showed how cultural and linguistic differences and organizational constraints limit health visitors’ ability to assess immigrant women's mental health needs. Although health visitors often used EPDS as an opener to talk about mental health, they did not use it as an ‘assessment’.

**Conclusions:**

This study shows inequalities in PPD screening than may result in reduced use of mental health services among immigrant women. The experiences of health visitors shed light on how to improve the identification of PPD among immigrant mothers.

**Key messages:**

• Migrant women in Denmark, even those who have migrated as children, are at risk of being left out of PPD screening programs.

• Limited organizational support and cultural competences makes PPD screening among immigrant women not feasible for maternal health visitors.

